# Case of Pregnancy, Notwithstanding Previous Severe Injury to the Organs Concerned in Childbirth

**Published:** 1850-08

**Authors:** 

**Affiliations:** Meeran, Schonburgh


### OBSTETRICS.

*Cases of Pregnancy, notwithstanding previous severe injury to the organs concerned in Childbirth.* By Dr. Leopold, of Meeran, Schonburgh.—*Case* 1.—Mrs. —— was delivered with instruments, on the 1st of January, 1843, by an experienced accoucheur, and suffered considerably from ischuria on the following day. On the 6th, Dr. Leopold saw her in consultation. A large hard tumor, tender to the touch, filled the greater part of the vagina. The nature of this tumor was doubtful, and continued so until the 9th inst., when, having taken a strong purgative, she felt during its action a substance pass from the vagina. Copious hæmorrhage occurred, followed by syncope. On examination the prolapsed and inverted uterus was found between her thighs, lying in a pool of blood, urine and fæces. It was immediately washed and returned. The hæmorrhage ceased; the fundus uteri could be felt beneath the abdominal parietes. The patient was confined to the recumbent posture for many weeks. She perfectly recovered her health, and was delivered of another child two years afterwards without instrumental aid. In this case it is possible that partial inversion of the uterus may have been occasioned at the time of delivery by adhesion of the placenta, and that the subsequent accident converted this into complete inversion and prolapsus.

*Case* 2.—Mrs. ——, the mother of five children, was in labor on March 30th, 1844. The arm presented, and it was necessary to turn. During the operation the patient exclaimed that she was suffering excruciating pain in the left side of the pelvis. She threw herself about in the most inconvenient positions just as Dr. Leopold had reached the foot. Much care was required to complete the delivery, as the pains also flagged. The placenta was expelled without hæmorrhage. At midnight of the same day Dr. Leopold was summoned to her, and found her lying with all the symptoms of severe hæmorrhage, but only a few ounces of blood were found in the bed. On passing the hand into the vagina several large coagula were expelled. The uterus was found lying over to the right side of the pelvis. The os uteri was open, and the uterus contained some coagula, which were quickly expelled by the contractions of the organ from external and internal stimulation. On the left side of the vagina, the hand entered a large sac full of blood. The orifice of this sac was about an inch from the os uteri, and was sufficiently large to admit the hand without force. Its walls were formed by the iliac bone, by the abdominal parietes, as high as the crista ilii, and by the iliacus internus muscle. A large quantity of coagulated blood was removed, and further hæmorrhage restrained by continued external application of cold. The patient refused all internal remedies. The prognosis was unfavorable. The extent of the injury indicated a remote origin; and, on inquiry, it was learnt that the patient had suffered from pain in the left side and hip during the whole period of her pregnancy, but had been compelled to follow her work at the loom.

The pains during labor were frequent and violent. A short time before the rupture of the membranes she had experienced a sensation as of a sudden and painful giving way of the parts on the left side. She had suffered from peritonitis after her previous confinement. A chronic abscess had no doubt existed in this spot, and was ruptured during labor by the force of the pains and the cross position of the child. The patient had again on this occasion an attack of puerperal fever, from which she recovered in three weeks. A purulent discharge continued for upwards of three months, after which she perfectly regained her health and strength, with the exception of diminished power in the left hip joint. Two years afterwards she was again confined, without any unfavorable occurrence.—*Lond. Med. Gaz.*

